# Ultrasound-Assisted Reverse Micelle Synthesis of ZnSe/ZnS Nanomaterials—Study of the Effect of Power and Water:Surfactant Ratio on Morphology and Crystallinity

**DOI:** 10.3390/molecules31172943

**Published:** 2026-08-22

**Authors:** Jaime Moroni Mora-Muñoz, Lorena Álvarez-Contreras, Luis A. Godínez, Luis J. Torres-Pacheco, Noé Arjona, Minerva Guerra-Balcázar

**Affiliations:** 1 Facultad de Ingeniería, División de Investigación y Posgrado, Universidad Autónoma de Querétaro, Queretaro C.P. 76010, Mexico; j.m.37296@gmail.com; 2 Centro de Investigación en Materiales Avanzados S.C., Complejo Industrial Chihuahua, Chihuahua C.P. 31136, Mexico; lorena.alvarez@cimav.edu.mx (L.Á.-C.); noe.arjona@cimav.edu.mx (N.A.); 3 Centro de Investigación en Química para la Economía Circular, CIQEC, Facultad de Química, Universidad Autónoma de Querétaro, Cerro de las Campanas, SN, Queretaro C.P. 76010, Mexico; luis.godinez@uaq.mx

**Keywords:** sonochemistry, laminar nanomaterials, pseudomorphic growth, reverse micelle, semiconductor heterostructures

## Abstract

The controlled formation of coherent interfaces in lattice-mismatched II–VI semiconductor heterostructures remains challenging. In this work, ZnSe/ZnS laminar nanomaterials were synthesized by an ultrasound-assisted reverse micelle method to determine how ultrasonic power (150 and 200 W) and water-to-surfactant molar ratio (16:1, 32:1, and 48:1) jointly regulate morphology, crystal structure, lattice accommodation, optical response, and photoelectrochemical behavior. Higher ultrasonic power favored more clearly defined laminar morphologies, whereas increasing the water-to-surfactant ratio produced more heterogeneous growth domains. XRD and Raman spectroscopy confirmed the presence of zinc-blende ZnSe and ZnS phases and provided indirect evidence consistent with partial pseudomorphic lattice accommodation, with calculated mismatch values of 1.59–3.07%, compared with the theoretical value of 4.43%. The apparent optical band gap decreased from 3.39 to 3.06 eV at 200 W and from 3.34 to 3.04 eV at 150 W as the water content increased. Photochronoamperometry showed predominantly cathodic responses, whereas P1R1 exhibited an anodic response. These results establish ultrasonic power and micellar composition as coupled synthesis parameters for tuning lamellar growth, interfacial strain, and optoelectronic response in ZnSe/ZnS heterostructures.

## 1. Introduction

The performance of semiconductor heterostructures is strongly governed by the interface between the coupled phases. In materials where the lattice parameters are different, uncontrolled growth of the crystalline phases may lead to lattice mismatch, strain relaxation, defects, and poor interfacial coherence, which can impact on the materials’ properties, such as their photoresponse.

**Scheme 1:**
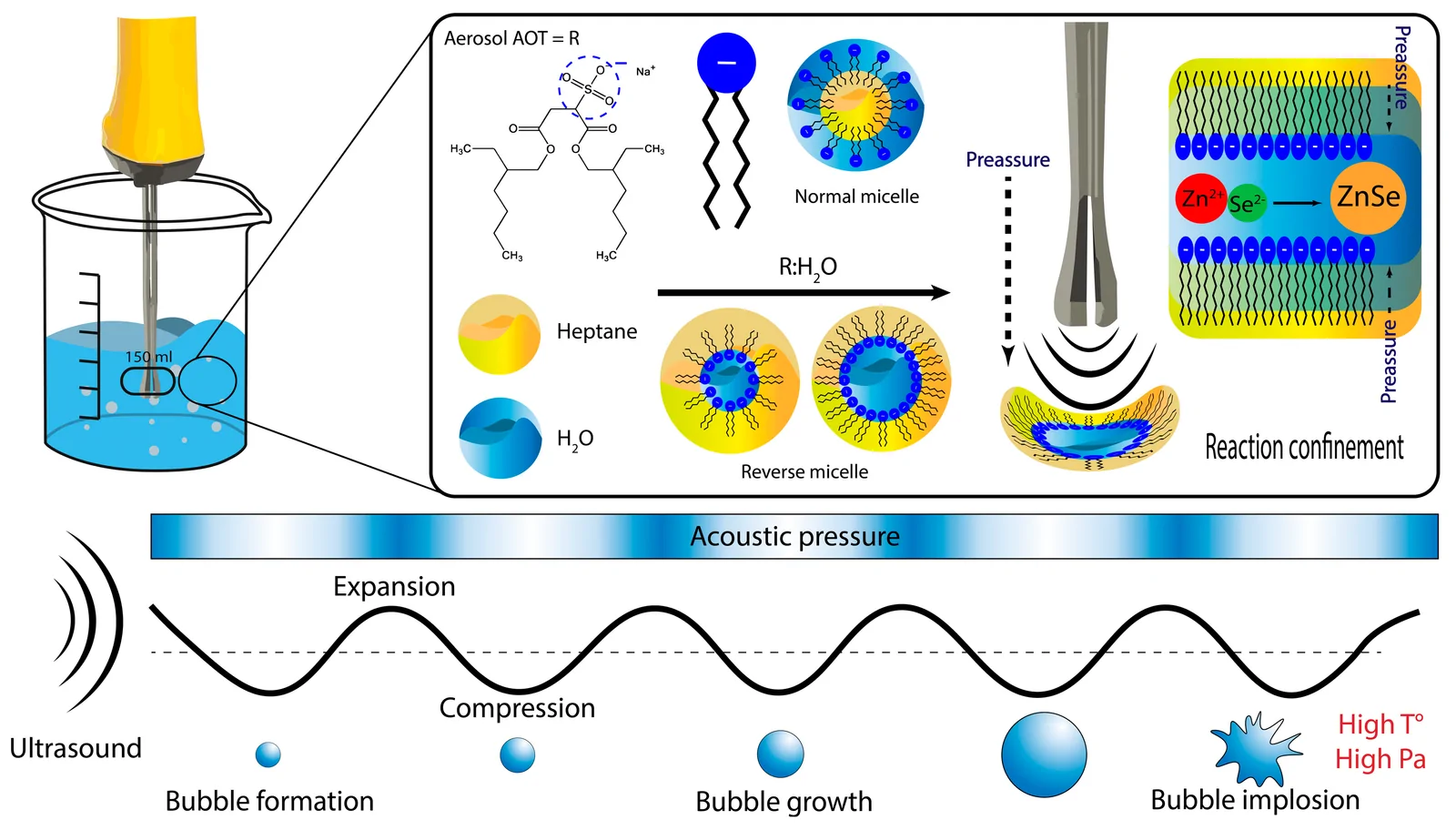
Synthesis of ZnSe via the ultrasound-assisted reverse micelle method.

The reverse micelle synthesis method of nanostructures involves the formation of water droplets stabilized by surfactants in a nonpolar solvent, creating a microreactor where the reaction can occur with precise control over particle size and distribution [1]. Alongside this method, sonochemistry has emerged as a versatile and powerful technique for the synthesis of nanomaterials [2]. As a consequence of ultrasonic stimulus, cavitation events in the reaction medium produce localized hot spots characterized by extremely high temperatures and pressures which in turn provide proper conditions for the confined formation of nano-structured materials (Scheme 1). This synthesis tool not only enables significant enhancement of reaction rate and yields, but also the production of materials characterized by specific structural features when properly coupled with other techniques [3]. In this regard, reverse micelle synthesis can be efficiently coupled with sonochemistry to offer a highly controlled environment for nanomaterial synthesis [4].

As can be readily anticipated, the simultaneous use of these two techniques defines essential operation parameters that must be properly understood in order to be successfully applied in standardized synthesis protocols: the phase ratio (water-to-surfactant ratio) and the ultrasound power. While ultrasonic power promotes energy concentration and mass transfer events through cavitation, reverse micelle structures modulate the microenvironment of the reaction medium, affecting the nanomaterial’s size, shape, and homogeneity [5,6]. The water ratio determines the stability and size of micelles, which in turn function as nanoreactors during the synthesis process: a higher water ratio can therefore result in larger micelles, leading to larger reactor size with potentially varied morphologies.

At constant frequency and under comparable reactor geometry, increasing the applied ultrasonic power generally increases the acoustic energy delivered to the medium and can intensify inertial cavitation, micromixing, mass transfer, and the generation of short-lived radical species. The effective sonochemical response nevertheless depends on duty cycle, probe position, dissolved gas content, temperature, and liquid composition. In AOT reverse micelles, increasing the water-to-surfactant ratio enlarges the aqueous core and changes the flexibility of the interfacial film and the exchange of precursor species between micelles. These changes can broaden the local nucleation and growth environments, favor secondary growth or aggregation, and reduce morphological uniformity. A dilution of short-lived radical species at higher water content may also contribute to less efficient precursor conversion; however, because radical concentrations were not measured in this work, this explanation is treated as a mechanistic hypothesis rather than a demonstrated process.

**Scheme 2:**
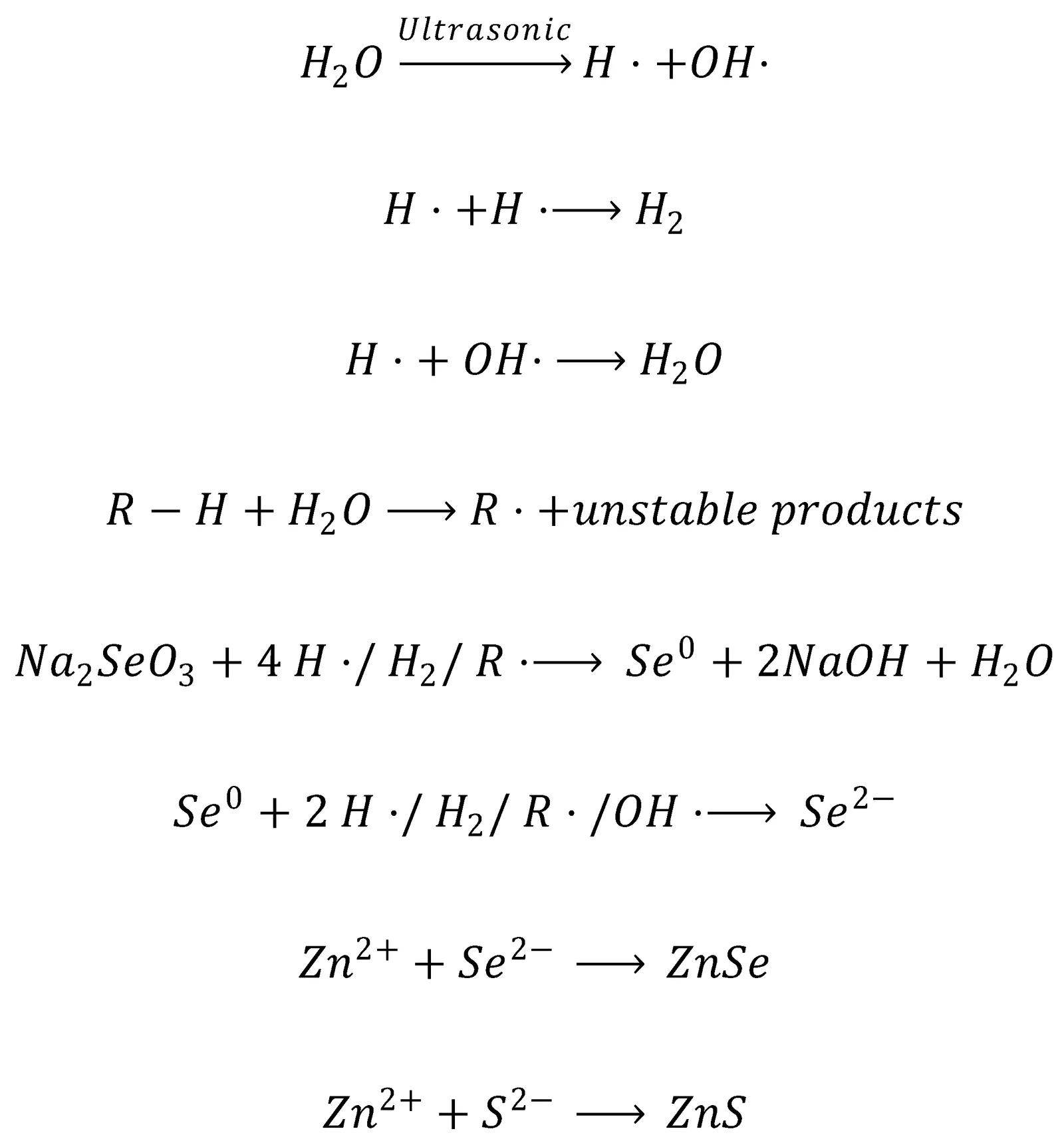
Radical chemical species generated during the cavitation process, and the formation of ZnSe/ZnS.

The coupling of the reverse micelle synthesis and sonochemistry methods enable two complementary effects: localized energy input through acoustic cavitation and spatial confinement within micellar nanoreactors. The cavitation effect promotes the formation of radicals, mass transfer and high-pressure and temperature domains (Scheme 2) [7,8], but more importantly, they effectively confine the reaction space to enhance reaction efficiency: the deformation of reverse micelles, induced by the high-pressure zones generated during ultrasonic cavitation, significantly contributes to this confinement, whereas reverse micelles restrict the reaction volume and modulate nucleation and growth. The combined phenomena should favor controlled ZnS growth over ZnSe by limiting uncontrolled aggregation [1].

ZnSe/ZnS nanomaterials are highly tunable with exceptional optical and electronic properties, making them ideal substrates for the study of the effect of synthesis conditions on their physicochemical properties [9]. Traditional synthesis methods for these materials, however, often encounter multiple limitations, such as the ZnS/ZnSe lattice mismatch and the lack of size and morphology homogeneity, leading to defects and suboptimal material performance [10]. ZnSe/ZnS was selected as a model heterostructure semiconductor system as the coupling of these II–VI phases allows the study of interfacial lattice accommodation, pseudomorphic growth, and defect formation. While ZnSe provides a narrower band gap phase, ZnS provides a wider band gap, which can act as a surface-passivating semiconductor phase, impacting on the optical properties of the resulting heterostructure semiconductor [10].

Conventional colloidal hot-injection and heating-up routes have been widely used to prepare ZnSe/ZnS core/shell quantum dots with controlled dimensions, shell thickness, and strong surface passivation [11,12]. These methods can provide narrow size distributions and high optical quality, but they typically employ high-boiling coordinating media, elevated temperatures, and carefully timed precursor addition. Hydrothermal approaches provide a different route to crystalline ZnS/ZnSe composites and allow crystallinity and photocatalytic behavior to be adjusted through temperature and reaction time, although the products are commonly particulate or aggregated and require prolonged thermal treatment [13]. The ultrasound-assisted reverse micelle approach investigated here addresses a different structural regime by combining confined aqueous nanoreactors with localized acoustic energy. Its main advantages are the independent control of acoustic power and aqueous core composition and the formation of lamellar architectures at comparatively low bulk temperature. Its limitations include morphology dispersion, residual surfactant-derived species, and incomplete conversion under some conditions.

The specific contribution of this work is therefore not limited to optimization of a single synthesis condition. The study establishes a comparative structure–property map linking ultrasonic power and water-to-surfactant ratio with projected lamellar dimensions, morphological heterogeneity, coherent domain size, lattice strain, semi-quantitative crystalline phase contributions, calculated lattice mismatch, apparent optical band gap, and photocurrent polarity. This combined analysis provides new insight into how acoustic energy and micellar confinement jointly regulate phase development and interfacial lattice accommodation in lamellar ZnSe/ZnS materials.

## 2. Results and Discussion

### 2.1. Morphology and Elemental Composition of Synthesized Laminar Materials

**Figure 1:**
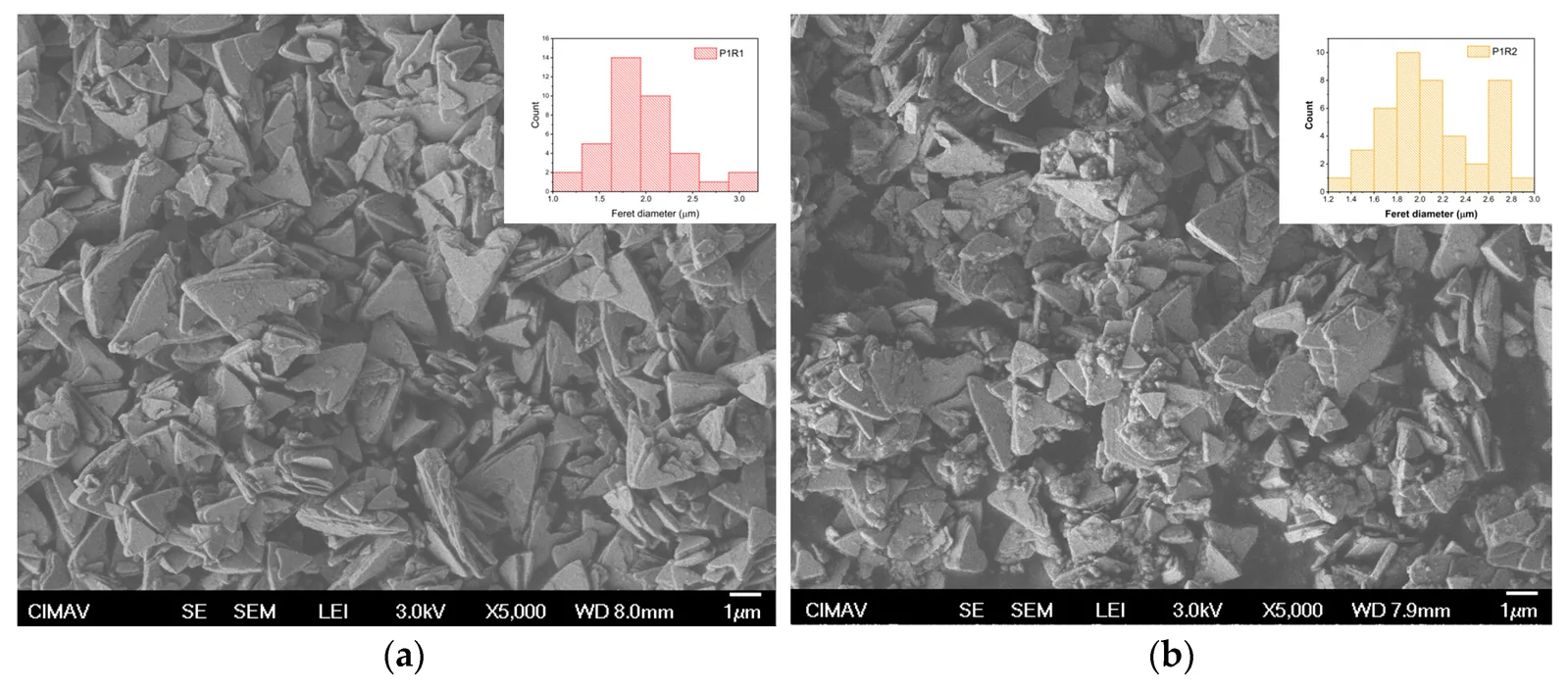

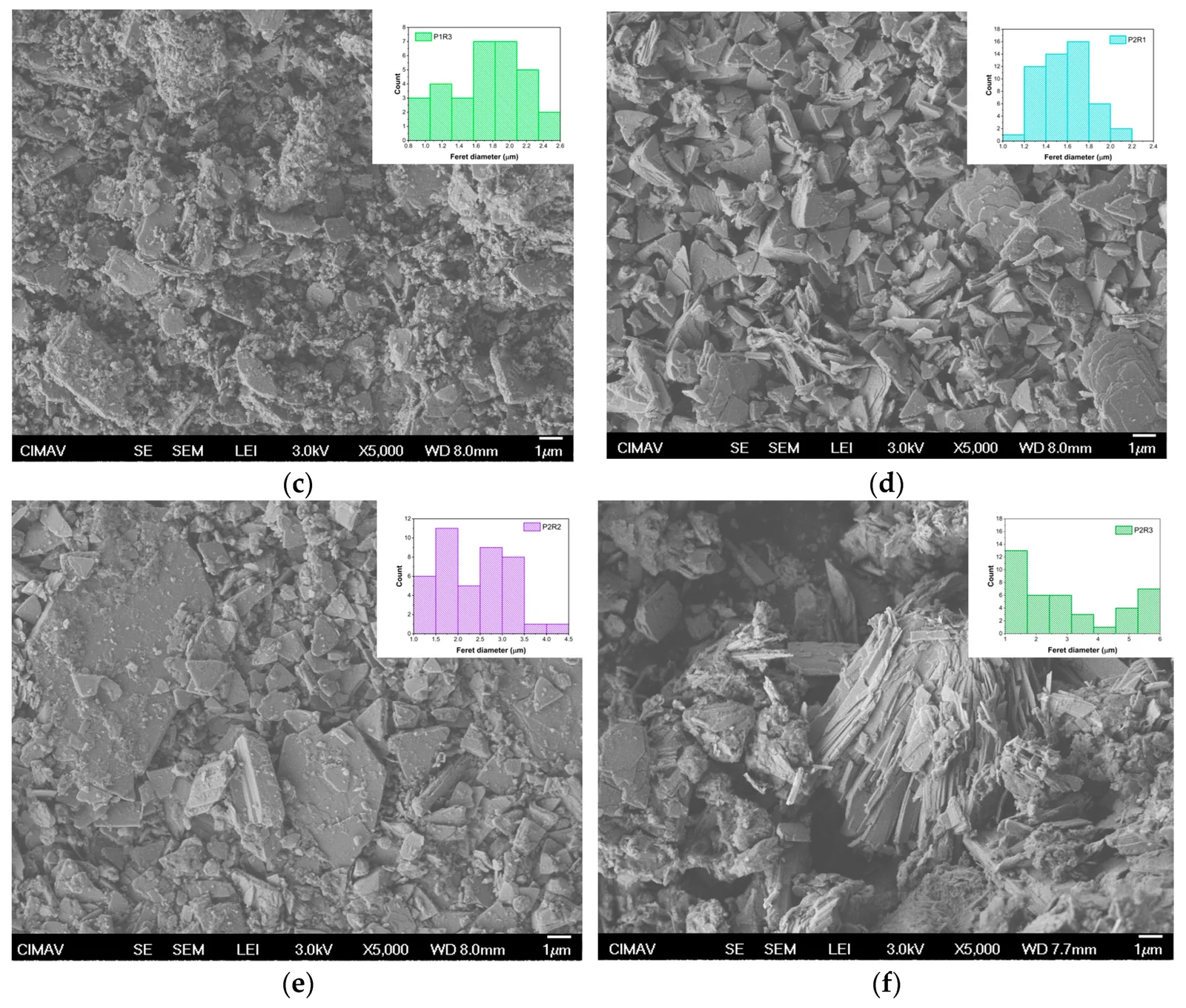
Scanning electron microscopy (SEM) of the synthesized materials by ultrasound-assisted reverse micelle method under different conditions: (**a**) P1R1, (**b**) P1R2, (**c**) P1R3, (**d**) P2R1, (**e**) P2R2, and (**f**) P2R3 with their respective Feret diameter histograms as insets.

The morphology of the synthesized materials was analyzed by scanning electron microscopy (SEM), as shown in Figure 1. The images of the materials synthesized at higher ultrasonic power, 200 W, revealed a predominant arrowhead-like laminar morphology for P1R1, a triangular laminar morphology for P1R2, and a non-homogeneous morphology for P1R3. For the materials synthesized at 150 W, P2R1 also exhibited arrowhead-like morphology, although smaller; this behavior can be attributed to the lower ultrasonic power employed, which may reduce the crystalline growth rate of the materials. However, additional secondary morphologies were also observed in both P1R1 and P2R1 samples. P2R2 also presented triangular laminar structures, but the lower ultrasonic power resulted in a higher contribution of secondary morphologies when compared with P1R2. Finally, P2R3 presented rectangular structures arranged in spherical-like aggregates, in contrast to P1R3, which did not show a well-defined morphology.

To provide a quantitative comparison while retaining the morphological complexity of the samples, maximum Feret diameters were measured for 252 discernible projected structures (*n*) across 12 SEM fields. Mean ± SD values were 1.94 ± 0.42 µm for P1R1 (*n* = 38), 2.19 ± 0.57 µm for P1R2 (*n* = 45), 1.84 ± 0.67 µm for P1R3 (*n* = 33), 1.58 ± 0.23 µm for P2R1 (*n* = 51), 2.60 ± 1.14 µm for P2R2 (*n* = 45), and 3.00 ± 1.59 µm for P2R3 (*n* = 40). P2R1 displayed the smallest mean projected dimension and the narrowest distribution, whereas P2R2 and P2R3 showed substantially broader distributions associated with the coexistence of different morphology classes and field-to-field heterogeneity. The effect of ultrasonic power was not monotonic across water-to-surfactant ratios: at R1, the mean maximum Feret diameter decreased from 1.94 µm at P1 to 1.58 µm at P2, whereas at R2 it increased from 2.19 to 2.60 µm and the dispersion doubled. Therefore, the measured dimensions reflect the combined effects of power, micellar water content, morphology class, stacking, and aggregation.

The obtention of laminar nanostructures is proposed to be the result of the flattening of the reverse micelles as consequence of the high-pressure zones formed due to the cavitation. This confines the reaction to these flattened micelles, resulting in the low-thickness nanostructures. Higher ultrasonic power (P1) resulted in more distinct and homogeneous morphologies compared to those with lower power (P2). These findings suggest that the increased energy input enhances cavitation events, promoting more pronounced structural features as well. Conversely, lower ultrasonic power led to less uniform and distinctive morphologies, indicating a less controlled cavitation process. The water-to-surfactant ratio (R) further modulates these effects by influencing micelle size and stability: larger micelles, associated with higher water content, produced larger and more varied nanoparticles.

To further study the synthesis methodology an elemental composition approximation of the ZnSe and the ZnS structures was obtained by energy dispersive spectroscopy (EDS) (Table 1). EDS analysis confirmed the presence of Zn and Se in the synthesized samples, while sulfur was detected at low atomic percentages in P1R1, P1R2, P1R3, and P2R1 [11]. C and O were also present in the samples due to impurities and synthesis subproducts [14]. The low S/Zn ratio suggests that ZnS is not acting as a ‘Shell’ over the material but rather as a thin surface-passivating phase; these results support the formation of ZnSe/ZnS structures with limited ZnS surface coverage instead of fully segregated ZnS particles [15].

EDS confirmed the presence of Zn and Se in all samples and detected sulfur at low atomic percentages in P1R1, P1R2, P1R3, and P2R1. Because AOT contains carbon, oxygen, and sulfur, residual surfactant-derived species may contribute to the C, O, and S signals. Oxygen-containing precursor residues, adsorbed surface species, and minor crystalline or amorphous oxygen-containing phases may also contribute. EDS does not distinguish chemical state or crystalline phase; therefore, the relatively high oxygen atomic percentages cannot be interpreted directly as ZnO content. The XRD area analysis identified only a small relative crystalline ZnO contribution (0.81–1.30%) and a minor ZnSeO_4_ contribution (4.74–5.06%). Carbon- and oxygen-containing surface species may nevertheless influence the local dielectric environment, surface state density, optical absorption, interfacial charge transfer, and electron–hole recombination. Their individual contributions cannot be isolated from the present EDS data and are therefore considered possible secondary influences.

### 2.2. Crystalline Structure Analysis

**Figure 2:**
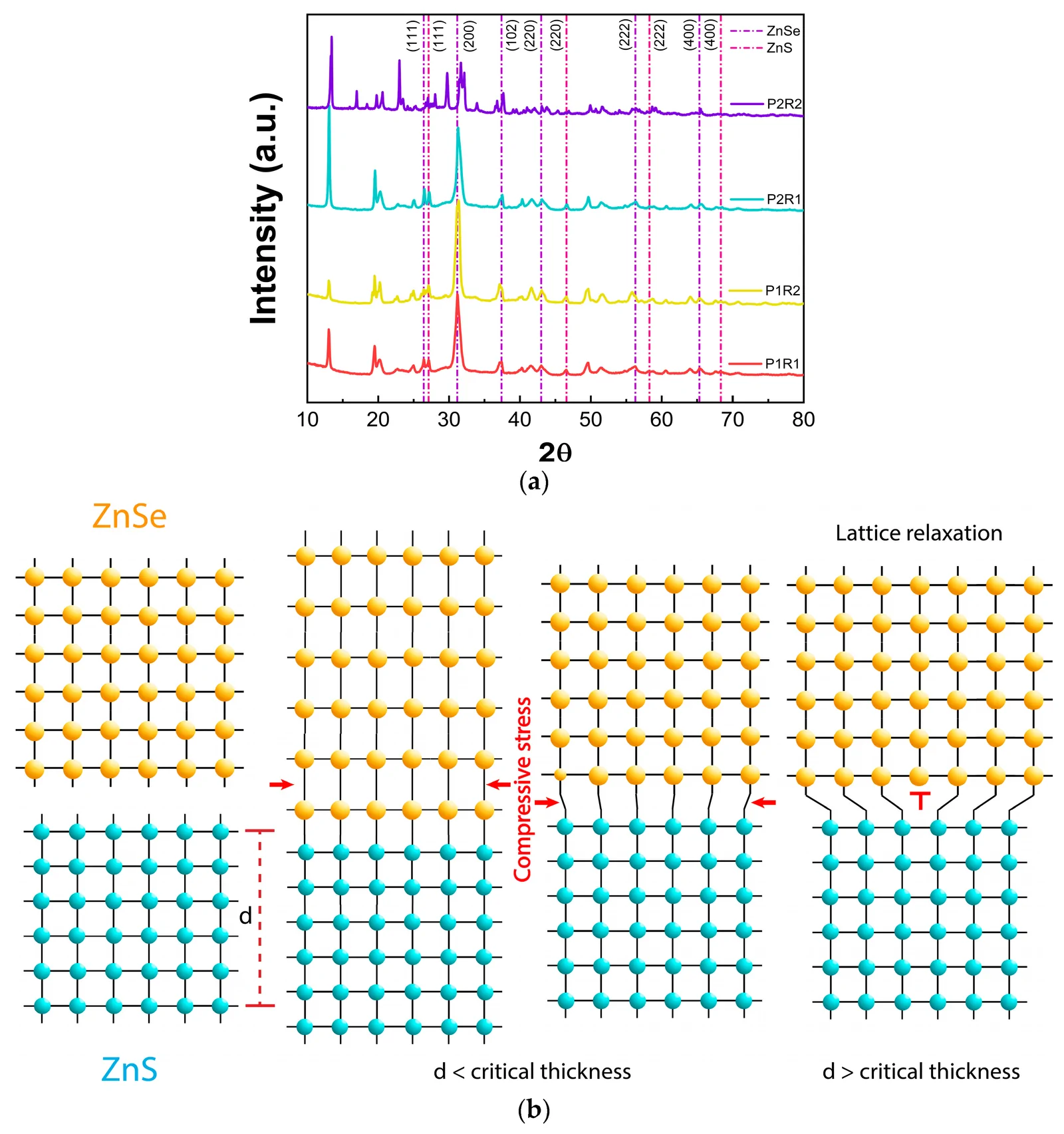
(**a**) Diffractograms of the ZnSe/ZnS material. (**b**) Diagram of superlattice formation in ZnSe/ZnS materials.

The synthesis methodology effect over the crystalline structure was studied by x-ray diffraction, focusing on the materials which exhibited a more homogeneous morphology, P1R1, P1R2, P2R1, and P2R2 (Figure 2a). The main diffraction reflections were assigned to zinc blende ZnSe and ZnS, while minor low-intensity reflections were associated with secondary phases such as Se, ZnSeO_4_, and ZnO [16,17,18]. These secondary reflections were considerably less intense than the characteristic ZnSe and ZnS diffraction signals, indicating that the predominant phases were those of these materials. The characteristic signals of ZnSe crystallographic planes correspond to 26.416° (111), 31.169° (200), 37.41° (102), 43° (220), 56.29° (222), and 65.32° (400) [19]; whereas ZnS planes were found at Bragg diffractions 27.12° (111), 46.56° (220), 58.27° (222), and 68.35° (400) [20]. The crystalline structure of the materials corresponds to zinc blende for ZnSe and ZnS and a preferential growth for ZnSe on plane (200) can also be observed. The corresponding diffraction signals for Se were found at 20.3°, 22.6°, 29.5°, and 51.5°; the corresponding Bragg diffractions to ZnSeO_4_ were located at 24.99°, 41.58°, 49.58°, and 64° [13,21]. To estimate the relative contribution of the indexed crystalline signals, the integrated net areas of the reflections assigned to each phase were summed and normalized to the total assigned diffraction area. ZnSe was the largest contribution in all samples, ranging from 69.89% in P1R1 to 43.48% in P2R2. The ZnS contribution ranged from 9.85 to 21.81%. Together, the intended ZnSe and ZnS phases accounted for 81.73% of the assigned area in P1R1, 78.53% in P1R2, 78.93% in P2R1, and 65.29% in P2R2. The relative Se contribution increased from 12.27% in P1R1 to 28.87% in P2R2, whereas ZnSeO_4_ remained approximately constant at 4.74–5.06% and ZnO remained low at 0.81–1.30%. The lower ZnSe contribution and higher residual Se contribution in P2R2 are consistent with less uniform phase development under the combined lower-power and higher-water condition. These values are semi-quantitative diffraction-area contributions and do not represent absolute phase fractions.

The Williamson–Hall method was used to determine the average crystallite size and lattice stress of the structures, and the crystallite size was also calculated by the Debye–Scherrer method for comparison [22] (Table 2). The crystallite size values obtained by the Williamson–Hall method were consistently higher than those calculated using the Debye–Scherrer equation, indicating that the peak broadening is not only associated with crystallite size but also with lattice strain. This distinction is relevant for ZnSe/ZnS heterostructures because the difference between the lattice parameters of both phases can generate strain during ZnS growth over the ZnSe structure. The lattice parameter of ZnS and ZnSe structures was calculated for a cubic structure for which the lattice parameter needed to be modified for a, b, and c [23]. The data obtained were compared with the corresponding theoretical values for ZnSe, 5.66 Å [24], and ZnS, 5.39 Å [25] (Table 3).

For ZnSe, the Williamson–Hall crystallite size increased from 19.61 nm for P1R1 to 43.32 nm for P2R2, whereas ZnS increased from 26.36 to 72.24 nm. This trend suggests that the water-to-surfactant ratio affects not only particle growth but also the ZnSe/ZnS interfacial region. Larger micellar nanoreactors may allow more extensive crystalline growth, although promoting morphological heterogeneity (Figure 1). The lattice strain values further indicate that the synthesized structures are not fully relaxed, supporting the presence of strain associated with interfacial accommodation between ZnSe and ZnS. The calculated lattice parameters were slightly higher than the theoretical values for both ZnSe and ZnS, suggesting lattice expansion associated with heterostructure formation. This deviation is particularly relevant because ZnS growth over ZnSe requires partial lattice accommodation to reduce interfacial mismatch. Therefore, the calculated parameters support the hypothesis that ZnS does not grow as a fully independent relaxed phase but rather adapts partially to the ZnSe lattice.

A paired comparison at equivalent water-to-surfactant ratios shows that ultrasonic power did not produce a monotonic change in coherent-domain size. At R1, the Williamson–Hall sizes for ZnSe and ZnS were 19.61 and 26.36 nm at P1, compared with 21.53 and 35.79 nm at P2. At R2, the corresponding values were 18.44 and 36.78 nm at P1 and 43.32 and 72.24 nm at P2. Thus, the largest coherent domains were obtained for P2R2 rather than for a higher-power sample. However, the combined relative ZnSe + ZnS diffraction-area contribution was higher for P1R2 (78.53%) than for P2R2 (65.29%), while the relative Se contribution increased to 28.87% in P2R2. These results suggest that lower power can permit the growth of larger coherent domains while simultaneously producing less uniform phase conversion or stronger phase segregation. Ultrasonic power therefore affects morphology, coherent domain growth, and phase development through different and non-monotonic mechanisms that are further modulated by micellar water content.

The calculated crystallographic parameters suggest the formation of superlattice-like ZnSe/ZnS heterostructures rather than a simple physical mixture of independent ZnSe and ZnS phases. This interpretation is supported by three observations: both phases exhibit zinc blende crystalline structure, the lattice parameters show partial deviation from their theoretical values due to lattice expansion, and the calculated mismatch between ZnSe and ZnS decreases compared with the theoretical mismatch. Together, these results suggest lattice accommodation at the ZnSe/ZnS interface [26]. These types of structures exhibit biaxial stress at the heterojunction interface due to the mismatch between the lattices of each compound. The lattice mismatch percentage can be calculated using Equation (1) [27]:

(1) $$F=\frac{\left[a_{0}\left(s\right)-a_{0}\left(f\right)\right]}{a_{0}(f)}\cdot 100,$$

where *a*_0_(*s*) and *a*_0_(*f*) correspond to the lattice parameters of the substrate (ZnSe) and the film (ZnS), respectively [28]. The calculated lattice mismatch percentage is presented in Table 4, where a decrease is observed, possibly indicating a lattice accommodation at the semiconductor interface. These data are further discussed in later parts of this work.

**Figure 3:**
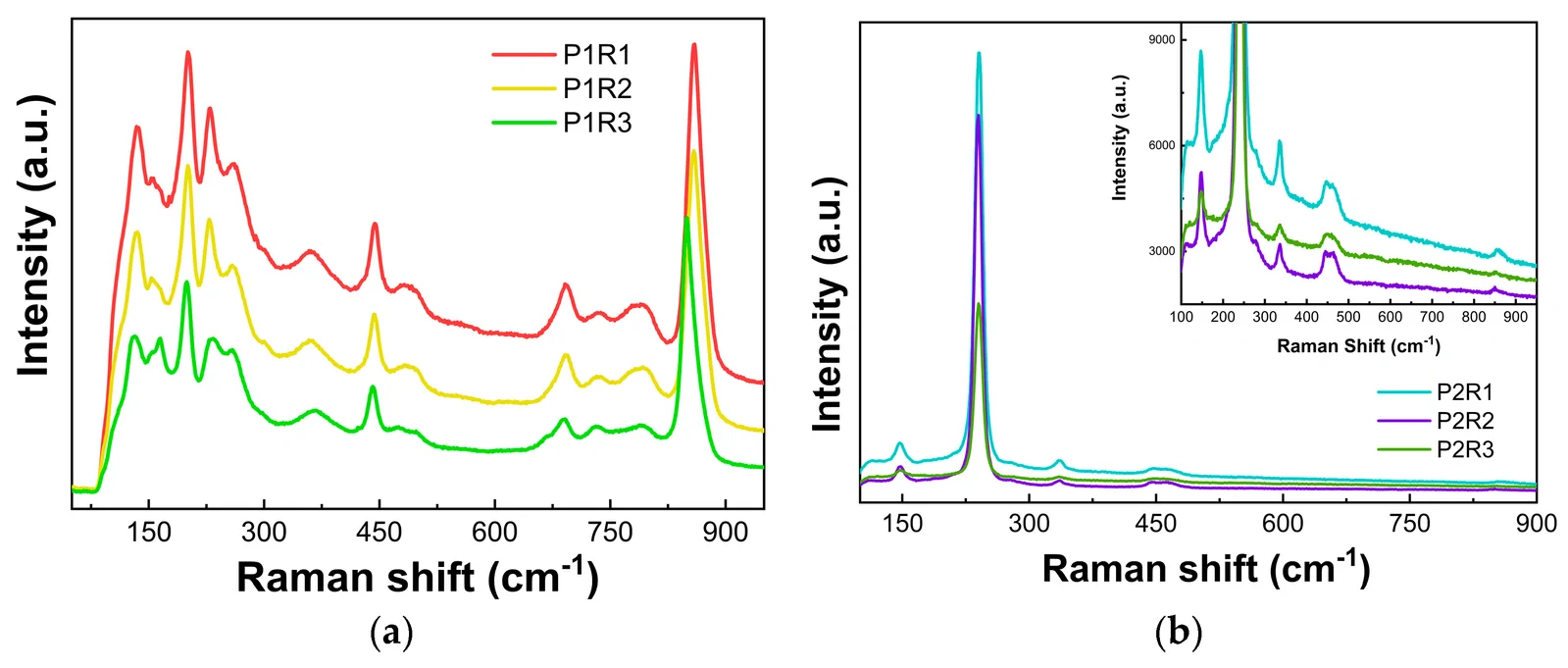
Raman spectra of ZnSe/ZnS synthesized at different ultrasonic powers: (**a**) P1RX materials. (**b**) P2RX materials. Inset: magnified view of the P2RX spectra at low intensity.

Raman spectroscopy was employed to gain deeper understanding of the pseudomorphic crystalline growth, providing additional insights into the crystalline structure and corroborating the zinc blende crystalline structure for ZnSe and ZnS [29,30] (Figure 3a,b). Vibrational modes processes involving more than one phonon are found in the range of 650–1000 cm^−1^, with some of these processes even exceeding single-phonon scattering as presented in the high-ultrasonic-power synthesized materials, P1 (Figure 3a). This suggests that the material’s morphology promotes interaction between several phonons, in which the processes of phononic transmission and radiation stand out. These phonon interactions occur typically through the scattering of phonons across the boundaries and interfaces between different materials, so that these interfaces can drastically affect the material’s properties [31]. In the range of 50–215 cm^−1^, it is possible to observe four predominant bands: 137 cm^−1^ (ZnSe 2TA(L), TO-TA(L)), 162 cm^−1^ (ZnSe LO-TA(L)), 176 cm^−1^ (ZnSe 2 T.A. (X) [32]), and 202 cm^−1^ (ZnSe TO [33]). From 215 to 300 cm ^1^ it is also possible to note two characteristic Raman bands located at approximately 230 cm^−1^ (ZnS TO [34]) and 260 cm^−1^ (ZnSe LO [33]). In the spectral region of 300–600 cm^−1^, two distinct Raman bands positioned around 362 cm^−1^ (ZnS LO [34]) and 445 cm^−1^ (ZnS LO + LA [35]) could be observed. In the 600–1000 cm^−1^ zone, there are four signals at 692 cm^−1^ (ZnS 2LO), 735 cm^−1^ (ZnS 2LO + TO [36]), 789 cm^−1^ (ZnS 3LO [37]), and 861 cm^−1^ (ZnSe 2LO + LO [38]). In these spectral zones, second- and third-order processes reveal the involvement of more than one phonon. These processes exhibit significant intensity when compared to the primary signals of the first-order modes, specifically the longitudinal optical modes of both ZnSe and ZnS.

In the Raman spectra of lower-ultrasonic-power synthesized materials, P2 (Figure 3b), a complete change in the spectrum can be observed, as most of the vibrational modes previously designated are altered. Based on these vibrational modes, the presence of the zinc blende crystal structure can be assumed to be similar for both materials. This synthesis group has no third-order vibrations with greater intensity than the first-order scattering modes. In the first zone of the Raman spectrum (50–600 cm^−1^), it is possible to observe the first distinct vibrational mode when compared to previous spectra, approximately at 147 cm^−1^ (Trigonal Selenium [34]). The primary vibrational mode of this material is also observed around 240 cm^−1^ (ZnSe L.O. [34]), confirming the correct formation of ZnSe. The formation of ZnS is observed through the ZnS vibrational modes present in the 300–600 cm^−1^ zone, near 335 cm^−1^ (ZnS L.O. [34]) and 446 cm^−1^ (ZnS LO+LA [32]). Similarly, a second-order vibration can be observed at approximately 464 cm^−1^ (ZnSe 2LO [34]). Only one third-order vibrational mode can be observed in the Raman spectrum for the P2 materials at approximately 854 cm^−1^ (ZnSe 2LO+LO [38]). However, this mode is not as intense as in previous structures. The lattice’s periodicity and crystal growth mechanisms cause the folding of the Brillouin zone center, leading to new phonon modes. If these modes comply with selection rules, they can be Raman-active [39].

With all Raman bands properly identified, the type of forces that are interacting in the material under study and the interaction between ZnSe and ZnS could be assessed. In this type of structure (superlattices), it is possible to evaluate the biaxial stress in the (001) plane through the optical vibrational modes using Equation (2) [40].

(2) $$\Delta \Omega^{LO}=\frac{\left[pS_{12}+q\left(S_{11}+S_{12}\right)\right]}{\omega_{0}^{LO}}X,$$

Here, S_11_ and S_12_ represent the elastic compliance constants, the parameters p and q are estimates derived under bulk material stress conditions, ω_0_ is the frequency of the bulk material’s vibrational modes under biaxial stress, and X is the applied stress [40]. Using reference values of the unperturbed modes (Table 5) it is possible to establish a relationship between the stress exerted due to the mismatch and the crystal lattices (Equations (3) and (4)). Calculated stress values are summarized in Table 6.

(3) $$\Delta \Omega_{ZnSe}^{LO}=-549.17\;X,$$

(4) $$\Delta \Omega_{ZnS}^{LO}=-733.07\;X,$$

The calculated values for the biaxial stress, through the optical longitudinal vibration of ZnSe and ZnS, suggest that the superlattice suffers compressive stress during formation [41]. In comparison with other computing methods, a higher degree of freedom during the growth of the second layer can be assumed for the materials under study. The stress data obtained for the lattice interface also suggests that, despite the difference between the lattice parameters of ZnSe and ZnS, there exists a synergic arrangement during the growth of the second layer. This indicates that at high ultrasonic power values, P1, the ZnS structure forms on top of the ZnSe structure without relaxation, thus producing a superlattice (Figure 2b), as can also be inferred from the residual stress present in the interface of these structures. This could be supported by the similarity of the biaxial stress data to which these two materials are subjected, which are similar in magnitude and sign (indicating compressive stress). This could indicate a pseudomorphic growth of ZnS on ZnSe and good coherence between ZnS and ZnSe. The difference in magnitudes may result from modifying the formed structure due to the stress they are subjected to, resulting in a slight curvature in morphology.

### 2.3. UV–Vis Spectroscopy

**Figure 4:**
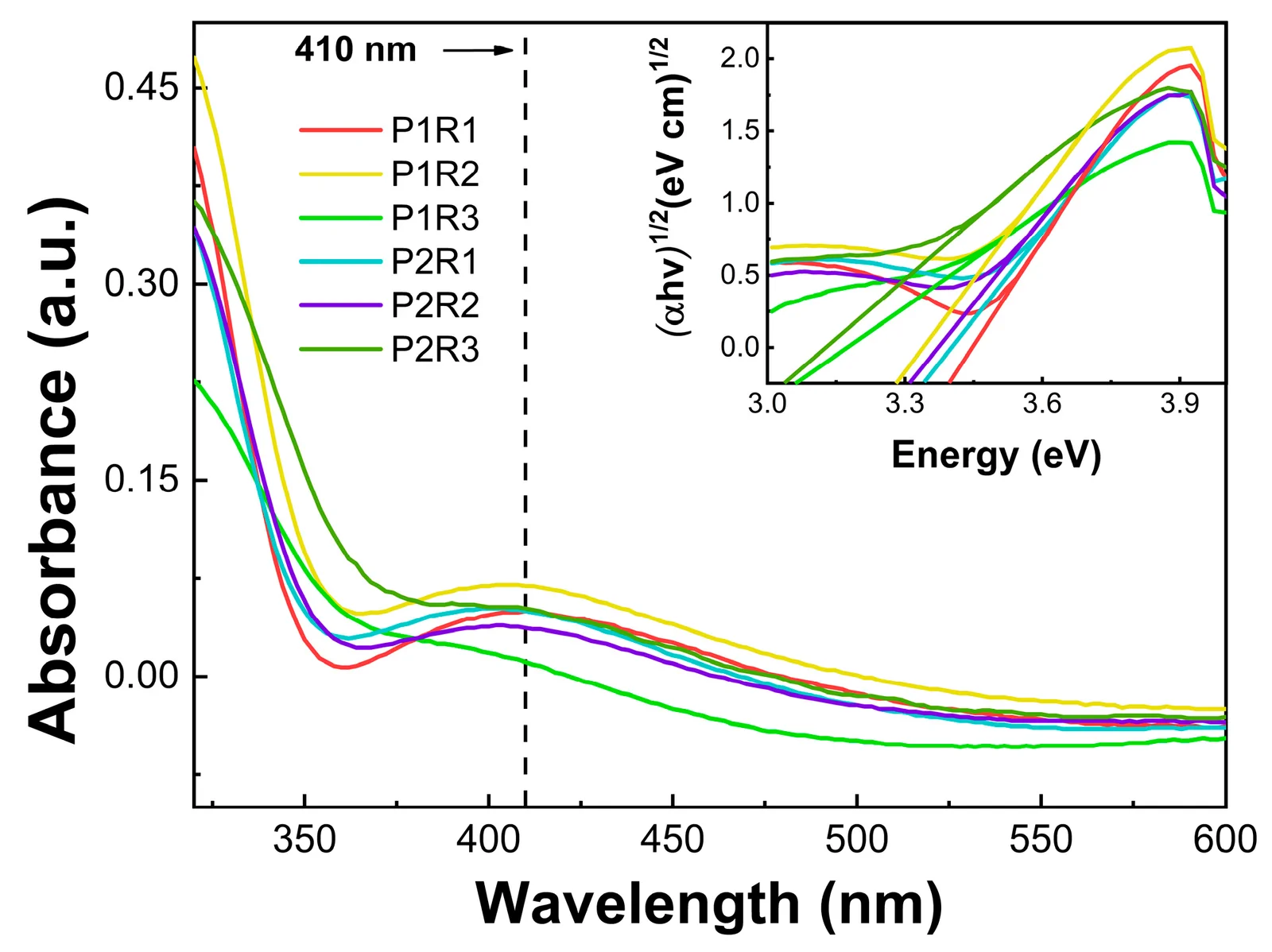
UV–Vis spectra of the nanomaterials, Inset: Tauc method.

UV–Vis spectroscopy was utilized to analyze the optical properties of the synthesized materials, such as the absorption characteristics and band gap energies, offering a comprehensive understanding of how the synthesis conditions affect the material’s optical behavior (Figure 4). The UV–Vis spectra exhibit an absorption signal around 420 nm, corresponding to ZnS/ZnSe [12]. The Tauc method was employed to determine the optical band gap of the material (Inset-Figure 4), resulting in values which are in good agreement with the reported ones: between 2.7 (ZnSe) and 3.7 eV (ZnS) [42,43]. UV–Vis spectroscopy was used to evaluate changes in the optical absorption behavior and apparent band gap energy of the ZnSe/ZnS heterostructures as a function of synthesis conditions. The absorption feature observed around 410–420 nm is consistent with the formation of ZnSe/ZnS semiconductor phases. The optical band gap, calculated using the Tauc method, decreased as the water-to-surfactant ratio increased. For P1 materials, the band gap decreased from 3.39 eV for P1R1 to 3.28 eV for P1R2 and 3.06 eV for P1R3. A similar trend was observed for P2 materials, decreasing from 3.34 eV for P2R1 to 3.31 eV for P2R2 and 3.04 eV for P2R3. This decrease suggests that higher water content promotes larger or more heterogeneous micellar environments, which may favor defect formation, lower crystalline order, and less coherent interfacial growth. These structural changes can introduce electronic states within the band structure, reducing the apparent optical band gap. Therefore, the optical response supports the structural evidence that micellar composition modulates the ZnSe/ZnS interface [44].

The combined results show that ultrasonic power and water-to-surfactant ratio affect different structural levels and should not be reduced to a single monotonic crystallinity trend. Higher ultrasonic power generally produced more clearly defined projected lamellar morphologies and, at R2, a larger combined relative ZnSe + ZnS diffraction area contribution. In contrast, lower power produced the largest coherent domains in P2R2 but also the highest relative Se contribution and a broad SEM size distribution, indicating that coherent domain growth does not necessarily imply more complete or homogeneous phase formation. Increasing the water-to-surfactant ratio enlarged the range of projected dimensions and promoted morphology- and field-dependent heterogeneity, particularly in P2R2 and P2R3. The progressive decrease in apparent optical band gap with increasing water content may therefore reflect a combination of phase distribution, residual species, surface states, morphology, and interfacial disorder. The available measurements support these correlations but do not isolate the individual electronic contribution of each factor.

Higher ultrasonic power enhances cavitation effects, promoting more efficient nucleation, improved mass transfer, and a more controlled growth front, which favors well-defined morphologies, higher crystallinity, and larger band gap values. This behavior suggests a lower contribution of defect-related electronic states and a more coherent ZnSe/ZnS interfacial arrangement. In contrast, increasing the water-to-surfactant ratio enlarges the reverse micellar nanoreactors and modifies their stability, leading to larger growth domains, more heterogeneous morphologies, and larger crystallites, but also to higher structural defects and less uniform interfacial growth. These effects are reflected in the progressive decrease in the band gap as the water-to-surfactant ratio increases, which may be associated with defect formation, reduced crystalline order, and changes in the electronic structure at the ZnSe/ZnS interface. Raman spectroscopy further supports this interpretation, since variations in vibrational modes indicate changes in crystallinity, lattice stress, and phonon propagation across the heterostructure. In the high-power P1 materials, the presence of more defined vibrational modes suggests greater long-range order, improved structural symmetry, and more stable vibrational coherence, whereas the P2 materials exhibit lower-intensity or absent modes, consistent with lower structural order and less coherent interfacial coupling. Therefore, the combined control of cavitation energy and micellar confinement demonstrates that ultrasound-assisted reverse micelle synthesis can be used as a tunable strategy to regulate morphology, crystallinity, lattice stress, band gap energy, and interfacial growth in ZnSe/ZnS semiconductor heterostructures [1].

### 2.4. Electrode Evaluation

As ZnSe/ZnS heterostructures are photoactive II–VI semiconductor materials, their functional performance is strongly influenced by charge carrier generation, separation, recombination, and transport across the semiconductor interface. Therefore, photoelectrochemical evaluation provides a useful functional probe to correlate interfacial growth, crystallinity, morphology, and surface states with the photocurrent response under UV illumination. In this context, photochronoamperometry was employed to assess whether the structural differences induced by ultrasonic power and water-to-surfactant ratio also affect the charge transport behavior.

**Figure 5:**
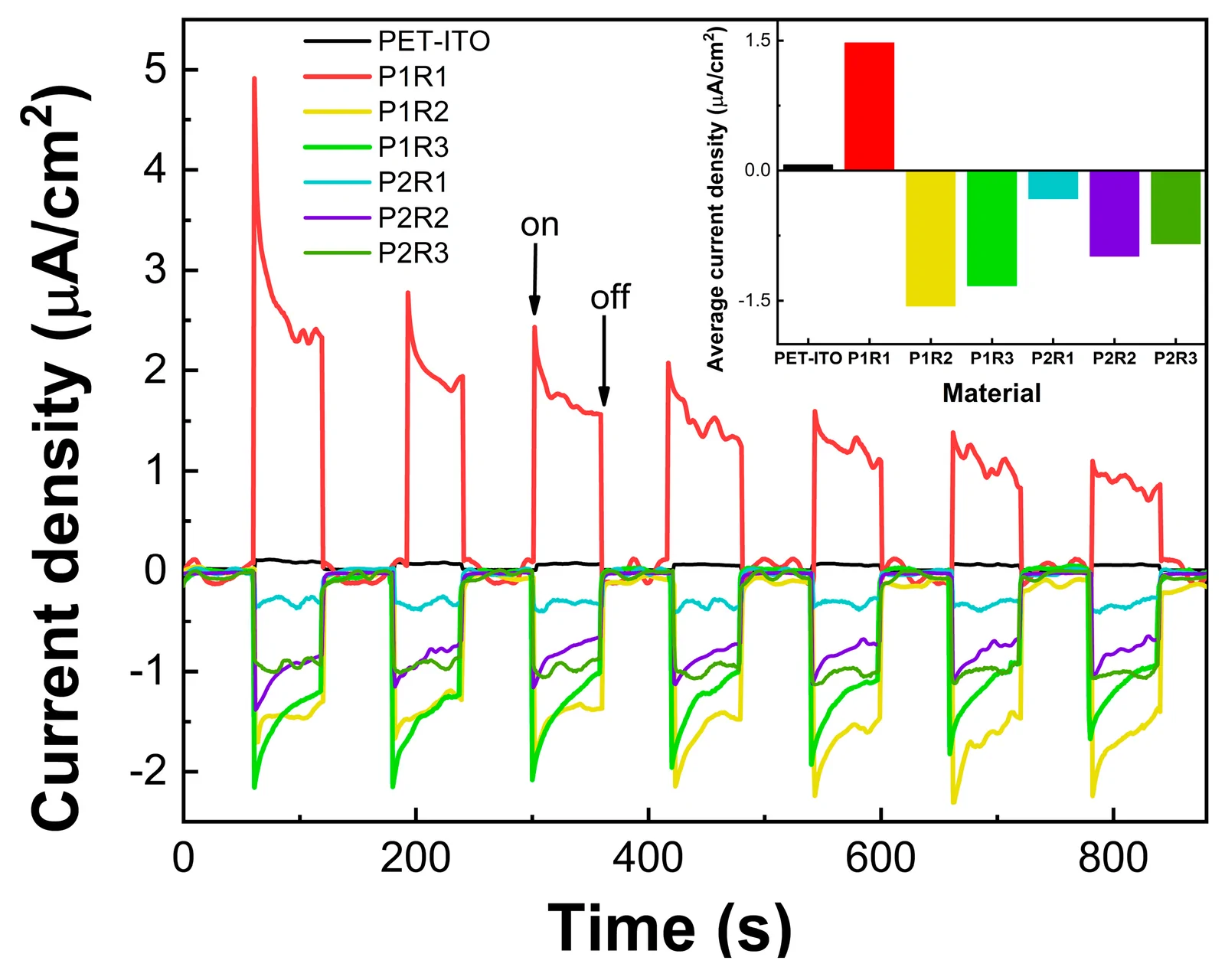
Photochronoamperometric response of PET-ITO-ZnSe/ZnS electrodes under intermittent 365 nm UV illumination. The inset shows the average photocurrent values obtained for each synthesized material.

A comprehensive assessment was conducted to evaluate how the structural and morphological features of the nanomaterials influence the photoelectrochemical performance of films prepared on conductive substrates and evaluated by chronoamperometry coupled with a 365 nm ultraviolet lamp (Figure 5). The electrodes exhibit a cathodic photocurrent associated with hole charge carrier transport [45], except for the P1R1 material, which presents an anodic photocurrent.

Upon illumination, a sharp increase in the photocurrent is observed, and it drops drastically almost immediately. Several factors participate in the recombination of photogenerated electron–hole pairs, depletion of reactants near the electrode surface, and changes in the electrode’s surface potential. The P1R1 material displayed an unusual anodic photocurrent, contrary to the anticipated cathodic behavior observed in other samples. The unusual anodic photocurrent observed for P1R1, in contrast to the cathodic response of the other samples, may be associated with differences in surface chemistry, interfacial trap states, or charge carrier dynamics produced by its particular morphology and crystalline structure. This interpretation is supported by the structural and compositional analyses, where lattice strain, partial lattice accommodation, low sulfur content, and the possible presence of residual or secondary species suggest that the ZnSe/ZnS interface is not fully ideal. These features can modify band bending, surface recombination, and the preferential direction of photogenerated charge transfer under UV illumination. This could lead to a reversal of the photocurrent from cathodic to anodic.

Additionally, the presence of impurities could affect the surface chemistry, creating surface states that alter the charge carrier dynamics and promote anodic behavior under UV illumination. The decrease in current observed after the first cycles can be attributed to material fatigue. Additionally, the material may undergo mechanical or chemical fatigue due to repeated cycles of illumination and darkness. This fatigue can lead to a relaxation of the crystalline structure, which alters the transport properties of the charge carriers, ultimately resulting in a decrease in photocurrent [46,47]. Nevertheless, additional analyses such as photoluminescence spectroscopy, electrochemical impedance spectroscopy, Mott–Schottky measurements, or transient photocurrent decay studies would be required to confirm the specific role of defect or trap states in the observed photoelectrochemical behavior.

The anodic photocurrent observed for P1R1, in contrast to the cathodic response of the other samples, may be related to differences in surface chemistry, residual species, interfacial trap states, charge transfer kinetics, or band bending associated with its morphology and structural characteristics. However, photochronoamperometry alone cannot distinguish among these possibilities and does not demonstrate band inversion. The observed polarity reversal is therefore reported as an experimental feature, whereas the proposed mechanisms remain tentative. Photoluminescence spectroscopy, Mott–Schottky analysis, electrochemical impedance spectroscopy, and transient carrier measurements would be required to determine the electronic origin quantitatively. Similarly, the progressive decrease in photocurrent amplitude during repeated illumination cycles may involve surface state filling, depletion of electroactive species near the film, photocorrosion, film restructuring, or changes in surface potential; the present data do not permit these contributions to be separated. The capacity to manipulate specific morphologies, crystallinities, and surface chemistries offers new possibilities for enhancing the performance of semiconductor nanostructures in a variety of technological applications.

## 3. Materials and Methods

### 3.1. Materials

Dioctyl sulfosuccinate sodium salt (AOT, >97%), zinc sulfate (ZnSO_4_, 99%), sodium selenite (Na_2_SeO_3_, 99%), sodium sulfide (Na_2_S, 99%), n-heptane (C_7_H_16_, 99%), and sodium sulfite (Na_2_SeO_3_, 99%) were obtained from Sigma–Aldrich, Inc. (St. Louis, MO, USA) and used as received. All aqueous solutions were prepared with distilled water, as well as all water sources employed.

### 3.2. Synthesis of ZnSe/ZnS Nanomaterials

The synthesis of ZnSe/ZnS was carried out through the following methodology. Three precursor solutions were prepared using a 0.1 M AOT solution in heptane. The water involved on each solution consisted of 0.3 M ZnSO_4_, 0.1 M Na_2_SeO_3_, and 0.1 M Na_2_S precursor solutions. Each solution was ultrasonically dispersed using an ultrasonic homogenizer, Hielscher UP200Ht (Hielscher Ultrasonics GmbH, Teltow, Germany) (200 W, 26 kHz) at 200 W for 5 min. The Zn and Se precursor solutions were mixed, and the ultrasonic homogenizer was then set to 10% cavitation duty cycle, for one hour. When the previous processes concluded, the S precursor solution was added, and the synthesis was kept under the same conditions for one hour. The resulting synthesized materials were classified by the synthesis power used (P1 = 200 W, P2 = 150 W) with respect to their water-to-surfactant molar ratios (R1= 16:1, R2 = 32:1, R3 = 48:1).

### 3.3. Electrode Fabrication

The electrodes were manufactured by means of electrophoresis of the synthesized powdered materials. The electrolyte employed was a 0.5 M NP2S and 0.1 M elemental sulfur aqueous solution with 1 mg of the material. The separation between the cathode and the anode was maintained at 1 cm. A 1 cm × 5.6 cm PET-ITO electrode was used as the cathode (where the material would be deposited), and a graphite electrode was used as the anode. The deposition potential and time corresponded to 1.5 V and 2 min, respectively. Afterwards the resulting electrodes were subjected to heat treatment at 60 °C for 12 h.

### 3.4. Physicochemical Characterization and Morphology Analysis

The morphology of the synthesized materials was studied using a scanning electron microscopy (SEM) instrument (JEOL JSM-7401 microscope; JEOL Ltd., Akishima, Tokyo, Japan). Two scanning electron microscopy fields were analyzed for each of the six synthesis conditions (P1R1, P1R2, P1R3, P2R1, P2R2, and P2R3). Each field was calibrated independently from its embedded 1 µm scale bar. Discernible structures with a complete projected contour, adequate separation for tracing, and no image boundary truncation were manually delineated in ImageJ version 1.54s. The elemental analysis of the materials was carried out using an energy-dispersive X-ray (EDX) spectrometer equipped with an EDX detector. X-ray diffraction patterns (XRD) were obtained using an X-ray diffraction instrument (Rigaku-ultima4; Rigaku Corporation, Akishima, Tokyo, Japan), utilizing a 1.5406 Å wavelength with a step size of 0.02 °/min. For the semi-quantitative phase area comparison, the net integrated areas of confidently assigned reflections were summed for each identified phase (ZnSe, ZnS, ZnSeO4, Se, and ZnO) and normalized to the sum of the integrated areas of all assigned phase reflections within the same sample. Using the Williamson–Hall method, patterns were employed to determine the crystalline structure of the synthesized materials and the remaining stress. For the Raman spectra, a HORIBA XploRA spectrometer (HORIBA FRANCE SAS, Palaiseau, France) equipped with a CCD sincerity detector and a 1200 gr mm^−1^ holographic slit, a 100 μm broad slit, and a confocal aperture of 300 μm was employed. The UV–Vis absorption spectra of the synthesized ZnSe/ZnS nanomaterial films deposited on the photocathodes were recorded using a Thermo Scientific Genesys 10S UV–Vis Spectrometer (Thermo Electron Scientific Instruments LLC, Madison, WI, USA). Electrochemical tests on the synthesized ZnSe/ZnS nanomaterials were conducted using an Autolab Potentiostat/Galvanostat PGSTAT-302N (Metrohm Autolab, Utrecht, The Netherlands) connected to a three-electrode electrochemical cell, in which the electrode under study, a saturated calomel, and a Pt wire were immersed in the solution and used as working, reference, and counter electrodes, respectively.

## 4. Conclusions

ZnSe/ZnS laminar semiconductor heterostructures were successfully synthesized by an ultrasound-assisted reverse micelle methodology, demonstrating that ultrasonic power and water-to-surfactant ratio govern material formation through their combined effects on cavitation energy and micellar confinement. Increasing the water-to-surfactant ratio enlarged the micellar reaction domains and promoted broader projected size distributions, mixed morphologies, stacking, and aggregation, particularly in P2R2 and P2R3, whereas lower water contents generally favored more defined laminar structures. The effect of ultrasonic power was not monotonic: higher power generally promoted better-defined morphologies, while lower-power P2R2 exhibited the largest coherent domains but also the broadest morphological distribution and the highest relative Se contribution, reaching 28.87%. This demonstrates that crystallite growth does not necessarily imply more complete or homogeneous ZnSe/ZnS formation. Depending on the synthesis condition, ZnSe and ZnS together accounted for 65.29–81.73% of the assigned diffraction area, while the calculated lattice mismatch decreased from the theoretical value of 4.43% to 1.59–3.07%. Therefore, ultrasonic power and micellar water content regulate interconnected structural features, including morphology, phase development, coherent domain growth, lattice strain, and partial interfacial accommodation.

These physicochemical variations were directly reflected in the optical and photoelectrochemical properties. Increasing the water-to-surfactant ratio progressively reduced the apparent optical band gap from 3.39 to 3.06 eV for the P1 series and from 3.34 to 3.04 eV for the P2 series, indicating that micellar composition could tune the electronic response through changes in morphology, phase distribution, surface states, residual species, and interfacial disorder. Photochronoamperometry showed predominantly cathodic behavior, whereas P1R1 exhibited an anodic response, further demonstrating that relatively small variations in the synthesis environment can modify photogenerated charge transfer pathways. Overall, the ultrasound-assisted reverse micelle method combines localized cavitation, enhanced mass transfer, and spatially confined micellar nanoreactors, providing a versatile route for tuning the structural and optoelectronic properties of laminar ZnSe/ZnS materials under comparatively mild conditions. The structure–property relationships established in this work provide a relevant basis for the future optimization of phase purity, morphological uniformity, and carrier dynamics, while supporting the development of photoelectrodes, UV-responsive devices, photocatalytic interfaces, and other semiconductor systems requiring tunable interfacial properties.

## Figures and Tables

**Table 1:** EDS analysis results for the synthesized 2D nanomaterials.

Sample	C at%	O at%	Se at%	S at%	Zn at%
P1R1	41.95	40.47	4.87	2.75	9.95
P1R2	19.07	57.02	9.59	1.83	12.48
P1R3	38.43	47.40	6.15	0.95	7.07
P2R1	21.62	58.70	7.25	1.27	11.17
P2R2	27.98	46.47	9.09	N.D.	16.46
P2R3	40.55	46.65	5.99	N.D.	6.81

N.D.: Not Detection.

**Table 2:** Results of the Debye–Scherrer and Williamson–Hall analysis for the synthesized 2D nanomaterials.

Material	Compound	Crystallite Size (Debye–Scherrer) (nm)	Crystallite Size (Williamson–Hall) (nm)	Lattice Strain (Williamson–Hall) ×10^−3^
P1R1	ZnSe	12.44	19.61	6.23
	ZnS	22.67	26.36	0.91
P1R2	ZnSe	12.39	18.44	7.52
	ZnS	26.54	36.78	1.61
P2R1	ZnSe	11.91	21.53	5.38
	ZnS	32.41	35.79	3.44
P2R2	ZnSe	33.05	43.32	5.75
	ZnS	64.23	72.24	1.34

**Table 3:** Computed lattice parameters for the 2D nanostructures crystal structures for ZnSe and ZnS.

Material	Compound	Average Lattice Parameter (Å)	Discrepancy Between Theoretical and Computed (%)
Theory	ZnSe	5.66	-
	ZnS	5.42	-
P1R1	ZnSe	5.71	0.876
	ZnS	5.59	3.041
P1R2	ZnSe	5.71	0.876
	ZnS	5.54	2.166
P2R1	ZnSe	5.69	0.527
	ZnS	5.53	1.989
P2R2	ZnSe	5.72	1.049
	ZnS	5.63	3.730

**Table 4:** Evaluation results for the lattice mismatch for the overlapping of ZnSe and ZnS crystalline structures.

Material	Compound	Average of Lattice Parameter (Å)	Mismatch Between Lattice Parameters (%)
Theoretical	ZnSe	5.66	4.43
	ZnS	5.42	
P1R1	ZnSe	5.71	2.15
	ZnS	5.59	
P1R2	ZnSe	5.71	3.07
	ZnS	5.54	
P2R1	ZnSe	5.69	2.89
	ZnS	5.53	
P2R2	ZnSe	5.72	1.59
	ZnS	5.63	

**Table 5:** Physical constants and Raman parameters used for biaxial stress calculation in ZnSe/ZnS heterostructures.

	ZnSe	ZnS
a (Å)	5.66	5.42
LO (cm^−1^) mode	252	349
$S_{11}$ (10^−12^ dyn^−1^cm^2^)	2.11	1.89
$S_{12}$ (10^−12^ dyn^−1^cm^2^)	−0.78	−0.72
p (10^28^ s^−1^)	−0.41	−0.39
q (10^28^ s^−1^)	−0.59	−0.99

**Table 6:** Raman band deviation and calculated biaxial stress values for ZnSe/ZnS laminar heterostructures.

**Deviation of the Raman Bands (cm^−1^)**						
**Material**	**P1R1**	**P1R2**	**P1R3**	**P2R1**	**P2R2**	**P2R3**
LO-ZnSe	8.14	9.39	9.39	−12.008	−12.008	−12.008
LO-ZnS	13.04	9.35	11.81	−14.081	−12.845	−12.845
**Stress on the Crystalline Lattice (MPa)**						
**Material**	**P1R1**	**P1R2**	**P1R3**	**P2R1**	**P2R2**	**P2R3**
LO-ZnSe	−1.482	−1.710	−1.710	2.187	2.187	2.187
LO-ZnS	−1.779	−1.276	−1.611	1.921	1.752	1.752

## Data Availability

The raw data supporting the conclusions of this article will be made available by the authors on request.
